# Solid–Electrolyte Interphase During Battery Cycling: Theory of Growth Regimes

**DOI:** 10.1002/cssc.202000867

**Published:** 2020-06-29

**Authors:** Lars von Kolzenberg, Arnulf Latz, Birger Horstmann

**Affiliations:** ^1^ Institute of Engineering Thermodynamics German Aerospace Center (DLR) Pfaffenwaldring 38–40 70569 Stuttgart Germany; ^2^ Helmholtz Institute Ulm (HIU) Helmholtzstraße 11 89081 Ulm Germany; ^3^ Ulm University (UUlm) Albert-Einstein-Allee 47 89081 Ulm Germany

**Keywords:** batteries, capacity fading, continuum modeling, lithium, solid–electrolyte interphase

## Abstract

The capacity fade of modern lithium ion batteries is mainly caused by the formation and growth of the solid–electrolyte interphase (SEI). Numerous continuum models support its understanding and mitigation by studying SEI growth during battery storage. However, only a few electrochemical models discuss SEI growth during battery operation. In this article, a continuum model is developed that consistently captures the influence of open‐circuit potential, current direction, current magnitude, and cycle number on the growth of the SEI. The model is based on the formation and diffusion of neutral lithium atoms, which carry electrons through the SEI. Recent short‐ and long‐term experiments provide validation for our model. SEI growth is limited by either reaction, diffusion, or migration. For the first time, the transition between these mechanisms is modelled. Thereby, an explanation is provided for the fading of capacity with time *t* of the form *t* ^*β*^ with the scaling coefficent *β*, 0≤*β*≤1. Based on the model, critical operation conditions accelerating SEI growth are identified.

## Graphical abstract

**Quo vadis?** The effect of how battery cycling affects solid–electrolyte interphase (SEI) growth rates is analyzed. To this aim, a continuum model of SEI growth is developed and compared to experiments on different time scales. With increasing time, the limiting mechanism changes from electron tunneling via reaction kinetics and charge diffusion to charge migration.

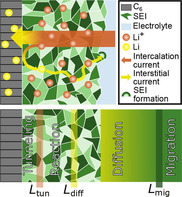

## Introduction

Lithium‐ion batteries are currently the state‐of‐the‐art portable energy storage devices as they provide high energy densities and long cycle lives. Increased battery lifetime and safety would promote the emergence of electromobility. However, continued capacity fade of lithium‐ion batteries remains an important challenge. The main cause of this capacity fade is the formation and growth of a solid–electrolyte interphase (SEI) on the graphitic anode.^1^, ^2^, ^3^, ^4^ Understanding the structure, composition, and continued growth of the SEI is thus key to extending battery life, improving battery safety, and developing new high‐energy electrodes.

The SEI is a thin layer that forms during the first charging cycle when the anode potential falls below the electrolyte reduction potential.^5^, ^6^, ^7^ Electrolyte molecules react with electrons and lithium ions, forming a nanometer‐thick layer of solids on the anode surface.^8^, ^9^ This layer protects the electrolyte from low anodic potentials in subsequent cycles, but the SEI continues to grow and consumes lithium ions in the process.

Different experiments have revealed that the SEI exhibits a dual‐layer structure with a dense inner layer and a porous outer layer. Inorganic compounds such as LiF, Li_2_CO_3_, and Li_2_O build up the inner layer and organic compounds such as dilithium ethylene dicarbonate (Li_2_EDC) build up the outer layer.^7^, ^10^, ^11^, ^12^, ^13^, ^14^, ^15^, ^16^, ^17^, ^18^, ^19^ Recent cryogenic electron microscopy measurements^20^, ^21^ give evidence that the different layers grow adjacent to each other on the particle surface. Some graphite particles are covered in a slowly growing dense SEI, while others are surrounded by a fast growing porous SEI. The experimental characterization of the underlying transport and reaction mechanisms is impeded by small length scales, air sensitivity, and the chemical variety of the SEI.

Electrochemical models give valuable complementary insights to reveal the role of the SEI within electrochemical cells.^6^, ^7^, ^22^ The macroscopic scale is discussed in a vast amount of literature with regard to voltage‐ and current‐dependent cell operation.^23^, ^24^, ^25^, ^26^ On the microscale, atomistic simulations were used to analyze the chemical structure, composition, and reactions of the SEI.^7^, ^27^, ^28^, ^29^ On the mesoscale, detailed continuum models shed light on the processes at the electrochemical interfaces.^30^, ^31^, ^32^ In these mesoscale models, it is well‐established that transport processes limit SEI growth during long‐term battery storage. Transport limitations lead to a capacity fade proportional to the square root of elapsed time, that is, √*t*. Different mechanisms were proposed to explain this behavior,^6^, ^33^ including solvent diffusion,^2^, ^30^, ^31^, ^32^, ^34^, ^35^, ^36^, ^37^, ^38^, ^39^, ^40^ electron conduction,^4^, ^30^, ^32^, ^37^, ^41^, ^42^, ^43^, ^44^ electron tunneling,^31^, ^36^, ^45^ and the diffusion of neutral lithium atoms.^18^, ^31^, ^46^ In a comparative study of these mechanisms, Single et al.^31^ identified neutral lithium diffusion as likely transport mechanism because it explains the state of charge dependence of the extensive storage experiments of Keil et al.^47^, ^48^

During battery operation, however, the external conditions, for example, charging rate and depth of discharge, strongly influence the SEI growth rate. The resulting capacity fade was analyzed in several papers with empirical formulas.^49^, ^50^, ^51^, ^52^, ^53^, ^54^, ^55^ These approaches nicely agree with experimental measurements but do not give further insights into underlying growth mechanisms. Physics‐based models for SEI growth during battery operation remain scarce and rely on solvent diffusion,^56^ electron conduction,^44^ or electron tunneling^45^ as charge‐transport mechanism.

In a recent joint experimental and theoretical work, the group of Bazant^44^, ^57^ investigated the influence of current, voltage, and cycle number on SEI growth. Attia et al.^57^ measured the capacity of carbon black *Q* over the voltage *V* during intercalation and deintercalation in their differential capacity d*Q*/d*V* experiments. They isolated the SEI contribution by comparing the second cycle with a high SEI contribution to a later baseline cycle with hardly any SEI contribution. Thereby, they revealed an asymmetry in SEI growth: During charging the SEI grows faster than during discharging. Das et al.^44^ modelled this asymmetry by assuming that the SEI is a mixed ion–electron conductor. In this model, the SEI conductivity depended on the concentration of lithium ions inside the SEI. The lithium ion concentration inside the SEI and hence the SEI formation current was high during charging and low during discharging. However, there were some inconsistencies in the modeling approach. First, recent models show that the SEI is a single‐ion solid electrolyte.^58^ Therefore, the lithium ion concentration inside the SEI should remain constant due to charge conservation. Second, the modeled conduction of electrons and lithium ions leads to counterpropagating fluxes. Thus, SEI formation should be fully suppressed during deintercalation. Third, the proposed mechanism of electron conduction disagrees with the electrode potential dependence of SEI growth observed in long‐term storage experiments.^47^, ^48^ Instead, the diffusion of radicals can explain these observations.^31^

In this paper, we discuss a consistent understanding of transport through the SEI and the dependence of SEI growth on operating conditions. The model consistently links the short‐term behaviour measured in the experiments of Attia et al.^57^ with the long‐term storage behavior measured by Keil et al.^47^, ^48^ For the first time, our approach shows the transition between different growth regimes, achieved by the coupling of the formation reaction and diffusion process of neutral lithium atoms in the SEI.

We present our model development in the next section and explain how it is implemented in the following section. Then, we validate the simulation using the differential capacity and capacity versus cycle number experiments of Attia et al.^57^ and show results for very long times. Then we make use of our model to analyze the influence of operating conditions on SEI growth with a focus on time dependence. Finally, we summarize the key findings of this work.

## Theoretical Background

**Figure 1:**
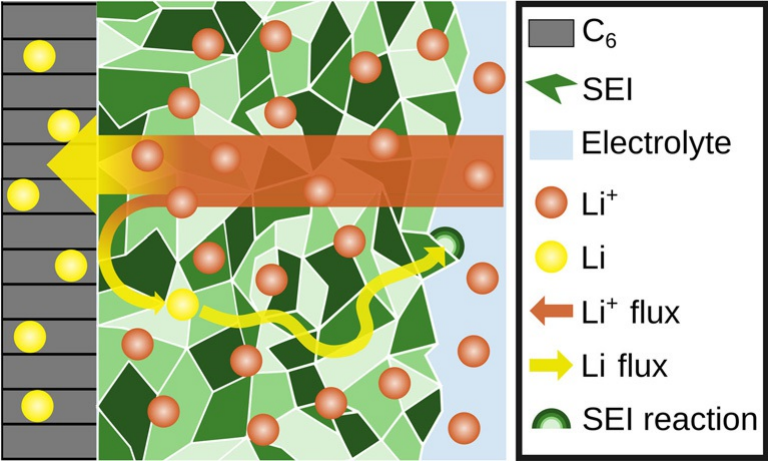
Schematic of the transport and reaction mechanisms in the SEI during battery charging. Neutral lithium atoms form at the electrode and move to the SEI–electrolyte interface by atom diffusion and electron hopping. Then they react with electrolyte and form fresh SEI. Lithium ions migrate through the SEI.

In this section, we present our theory for SEI growth based on the concept depicted in Figure 1.

At the electrode–SEI interface, lithium ions (Li+SEI ) from the SEI react with electrons (e^−^) from the electrode. The resulting neutral lithium atoms either intercalate into the electrode in the form of Li_*x*_C_6_ [see Eq. (1)] or remain as neutral lithium atoms (Li^0^) inside the SEI [see Eq. (2)],

(1) xLi+SEI+xe-+C6←→LixC6,

(2) Li+SEI+e-←→Li0

The neutral lithium atoms Li^0^ subsequently move through the SEI to the SEI–electrolyte interface, where they immediately react and form new SEI. According to reaction Equations (1) and (2), the overall measured current density of electrons (*j*) consists of the intercalation *j*_int_ and the SEI formation current density (*j*_SEI_),

(3) $$j=j{}_{\mathrm{int}}+j{}_{\mathrm{SEI}}$$

First, we will discuss the equations for transport of neutral lithium atoms Li^0^. Afterwards, we will derive an expression for the kinetics of lithium intercalation and neutral lithium atom formation. Finally, we combine the formation and transport currents of neutral lithium atoms to obtain an expression for the SEI growth current density (*j*_SEI_) and the resulting SEI thickness (*L*_SEI_(*t*)).

### Transport of neutral lithium atoms

We divide the electron transport from the electrode–SEI to the SEI–electrolyte interface into two contributions. First, the electrons tunnel a distance *L*_tun_ into the SEI and react to Li^0^, according to Equation (2). Second, the electrons move as neutral lithium atoms Li^0^ to the SEI–electrolyte interface. We account for the tunneling process by introducing an apparent SEI thickness (*L*_app_).

(4) $$L{}_{\mathrm{app}}=L{}_{\mathrm{SEI}}-L{}_{\mathrm{tun}}$$

Electrons can either move together with a neutral lithium atom or hop between lithium ions. For both cases, we use the dilute solution theory^59^ to model the transport current density *j*_SEI_,

(5) $$j_{\mathrm{SEI}}=\underset{\mathrm{diffusion}}{\underbrace{-z_{e^{-}}FD\nabla c_{\mathrm{Li}}}}-\underset{\mathrm{electromigration}}{\underbrace{\frac{z_{e^{-}}^{2}DF^{2}}{RT}c_{\mathrm{Li}}\nabla \phi }}$$

with the diffusion coefficient *D* of neutral lithium atoms [m^2^ s^−1^] and the concentration *c*_Li_ of neutral lithium atoms inside the SEI. Here, *F* is Faraday's constant (96485 C mol^−1^), *R* the universal gas constant (8.31 J mol^−1^ K^−1^), and *T* the temperature [K]. We assume isothermal operation as we focus on the electrochemistry of SEI growth and discuss the experiments of Attia et al.^57^ The electromigrative part of the flux describes electron transport due to an external electric field and depends on the valency of an electron *z* ${}_{e{}^{-}}$ =−1 and the electrical potential ϕ in the SEI.

We linearly approximate the gradients along the diffusion–migration path *L*_tun_≤*L*≤*L*_SEI_. We assume that electrons reaching the electrolyte are directly consumed to form a new SEI, so that *c*_Li_(*x*=*L*_SEI_)=0.^31^ Accordingly, the average concentration of neutral lithium atoms inside the SEI is *c̄*_Li_=*c*_Li_(*x*=*L*_tun_)/2. Using these assumptions and simplifications, we express the SEI current with Equation (6),

(6) $$j_{\mathrm{SEI}}=-DF\frac{c_{\mathrm{Li}}\left(L_{\mathrm{tun}}\right)}{L_{\mathrm{app}}}\left(1+\frac{F}{2RT}(\phi (L_{\mathrm{SEI}})-\phi (L_{\mathrm{tun}}))\right).$$

Ohm's law gives an expression for the potential difference in Equation (6),

(7) $$\phi \left(L_{\mathrm{SEI}}\right)-\phi \left(L_{\mathrm{tun}}\right)=-\frac{L_{\mathrm{app}}}{\kappa_{Li^{+},\mathrm{SEI}}}j_{\mathrm{int}},$$

with the lithium ion conductivity of the SEI *κ* ${}_{\mathrm{Li}{}^{+},\mathrm{SEI}}$ . By inserting Equation (7) into Equation (6), we obtain our final description of the diffusive–migrative electron current density through the SEI,

(8) $$\begin{array}{cc} j_{\mathrm{SEI}} & =-\frac{c_{\mathrm{Li}}\left(L_{\mathrm{tun}}\right)DF}{L_{\mathrm{app}}}\left(1-\frac{F}{2RT}\frac{L_{\mathrm{app}}}{\kappa_{Li^{+},\mathrm{SEI}}}j_{\mathrm{int}}\right) \end{array}.$$

### Intercalation

We describe the intercalation current density (*j*_int_) resulting from Equation (1) using a standard Butler–Volmer approach^59^, ^60^, ^61^

(9) $$j_{\mathrm{int}}=2j_{0}\sinh\left(\frac{F}{2RT}\eta_{\mathrm{int}}\right).$$

The overpotential (*η*_int_) for Equation (1) is defined by Equation (10),

(10) $$\eta_{\mathrm{int}}=\phi_{S}-U_{0}-\mu_{Li^{+},\mathrm{SEI}},$$

with the electrode potential $\phi_{S}$ , the open‐circuit voltage (OCV; *U*_0_), and the electrochemical potential of lithium ions at the electrode–SEI interface (*μ* ${}_{\mathrm{Li}{}^{+},\mathrm{SEI}}$ ). Accordingly, *η*_int_ and *j*_int_ are negative for intercalation and positive for deintercalation. The consistent exchange current density *j*_0_, defined by Equation (11),

(11) $$j_{0}=j_{0,0}\sqrt{\frac{c_{s}}{c_{s,\max}}}$$

depends only on the lithium concentration inside the electrode (*c*_s_) relative to the maximum concentration (*c*_s,max_). We assume that the lithium ion concentration inside the SEI *c* ${}_{\mathrm{Li}{}^{+},\mathrm{SEI}}$ is constant because the SEI is a single‐ion solid electrolyte with a fixed amount of charge carriers due to charge neutrality.^31^ Thus, the exchange current density *j*_0,0_ does not depend on *c* ${}_{\mathrm{Li}{}^{+},\mathrm{SEI}}$ . The *c*_s_ in the carbon black electrode changes with time according to Equation (12),

(12) $$\frac{dc_{s}}{dt}=-\frac{A_{\mathrm{cb}}}{F}j_{\mathrm{int}},$$

where *A*_cb_ is the volume‐specific surface area of carbon black.

### Formation reaction of neutral lithium atoms

SEI growth could be limited by two reactions, either neutral lithium atom formation at the electrode–SEI interface or electrolyte reduction at the SEI–electrolyte interface. Here, we present a simplistic model to shed light on the basic principles. Thus, we take into account only the kinetics of neutral lithium atom formation [see Eq. (2)]. We describe these reaction kinetics with an asymmetric Butler–Volmer approach,^59^, ^60^, ^61^

(13) $$j_{\mathrm{SEI}}=j_{\mathrm{SEI},0}\cdot \left(e^{\left(1-\alpha_{\mathrm{SEI}}\right)\frac{F\eta_{\mathrm{SEI}}}{RT}}-e^{-\alpha_{\mathrm{SEI}}\frac{F\eta_{\mathrm{SEI}}}{RT}}\right).$$

We choose as asymmetry factor *α*_SEI_=0.22 in line with the density functional theory results of Li and Qi^62^ and the microfluidic test‐cell measurements of Crowther and West.^63^ The Li^0^ formation overpotential (*η*_SEI_) in Equation (13) follows from the reaction Equation (2) as

(14) $$\eta_{\mathrm{SEI}}=\phi_{S}-\mu_{Li^{+},\mathrm{SEI}}+\mu_{\mathrm{Li}}/F.$$

We determine the chemical potential *μ* of the neutral lithium atoms with a dilute‐solution approach,^59^

(15) $$\mu_{\mathrm{Li}}=\mu_{\mathrm{Li},0}+RT\ln\left(\frac{c_{\mathrm{Li}}}{c_{\mathrm{Li},0}}\right).$$

The chemical potential assumes its standard value (*μ*_Li,0_) relative to lithium metal if the neutral lithium atom concentration at the electrode–SEI interface (*c*_Li_) equals the reference concentration of *c*_Li,0_=1 mol L^−1^. The exchange current density (*j*_SEI,0_),

(16) $$j_{\mathrm{SEI},0}=j_{\mathrm{SEI},0,0}{\left(\frac{c_{\mathrm{Li}}}{c_{\mathrm{Li},0}}\right)}^{\alpha_{\mathrm{SEI}}},$$

depends on the neutral lithium atom concentration at the electrode *c*_Li_, as we assume a constant lithium ion concentration inside the SEI.

We couple battery operation to Li^0^ formation by rephrasing Equation (13). Combining Equations (13)–(16), we obtain the following expression for the Li^0^ formation kinetics,

(17) $$j_{\mathrm{SEI}}=j_{\mathrm{SEI},0,0}\cdot \left(\frac{c_{\mathrm{Li}}\left(L_{\mathrm{tun}}\right)}{c_{\mathrm{Li},0}}e^{\left(1-\alpha_{\mathrm{SEI}}\right){\tilde{\eta }}_{\mathrm{SEI}}}-e^{-\alpha_{\mathrm{SEI}}{\tilde{\eta }}_{\mathrm{SEI}}}\right).$$

The dimensionless overpotential for neutral lithium atom formation ($\tilde{\eta }$_SEI_) follows from combining Equations (14), (15), and (10). This yields

(18) $${\tilde{\eta }}_{\mathrm{SEI}}=\frac{F}{RT}\left(\eta_{\mathrm{int}}+U_{0}+\mu_{\mathrm{Li},0}/F\right),$$

as a function of the OCV and *η*_int_, which depends on *j*_int_ according to Equation (9).

### SEI growth rates

So far, we derived expressions for the diffusive–migrative current density through the SEI [Eq. (8)] and the SEI growth based on the formation kinetics of neutral lithium atoms [Eq. (17)]. However, we do not know the current‐ and voltage‐dependent concentration of neutral lithium atoms (*c*_Li_(*L*_tun_)) inside the SEI. The two unknowns *j*_SEI_ and *c*_Li_(*L*_tun_) are determined by the two Equations (17) and (8). This results in Equation (19) for SEI growth (“+” for intercalation, “−” for deintercalation),

(19) $$j_{\mathrm{SEI}}=-j_{\mathrm{SEI},0,0}e^{-\alpha_{\mathrm{SEI}}{\tilde{\eta }}_{\mathrm{SEI}}}\frac{1\pm \frac{L_{\mathrm{app}}}{L_{\mathrm{mig}}}}{1\pm \frac{L_{\mathrm{app}}}{L_{\mathrm{mig}}}+\frac{L_{\mathrm{app}}}{L_{\mathrm{diff}}}}.$$

Note that this is an implicit equation for *j*_SEI_ as $\tilde{\eta }$_SEI_ depends on *j*_SEI_ through *η*_int_ [see Eq. (18)]. In Equation (19), *L*_diff_ and *L*_mig_ are the critical thicknesses for diffusion and migration, respectively. They are defined by

(20) $$L_{\mathrm{diff}}=\frac{c_{\mathrm{Li},0}DF}{j_{\mathrm{SEI},0,0}}e^{-\left(1-\alpha_{\mathrm{SEI}}\right){\tilde{\eta }}_{\mathrm{SEI}}},$$

(21) $$L_{\mathrm{mig}}=\frac{2RT\kappa_{Li^{+},\mathrm{SEI}}}{F\left|j_{\mathrm{int}}\right|}.$$

**Figure 2:**
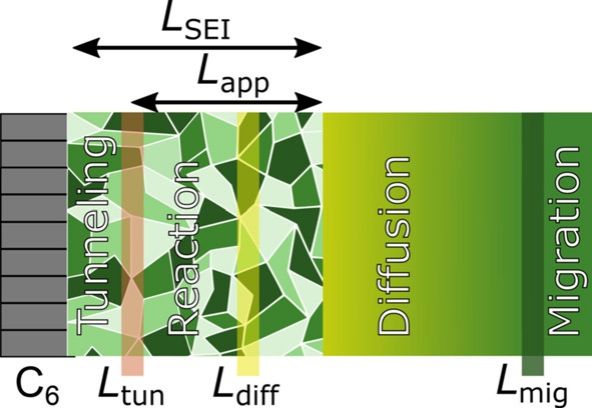
Schematic of the different thicknesses and the corresponding growth‐limiting steps. For a charging current density of *j*=C/5 and an OCV of *U*_0_=0.01 V our choice of parameters (see Supporting Information (SI), Table SI‐1) yields *L*_tun_=2.05 nm, *L*_diff_=25 nm, *L*_mig_=620 nm.

For realistic parameters, *L*_diff_≪*L*_mig_ holds (see Supporting Information, Table S1). We schematically summarize the different characteristic lengths in Figure 2.

We assume that each electron reaching the SEI–electrolyte interface is instantly consumed by SEI formation. Thus, we link the SEI current *j*_SEI_ directly to the SEI growth rate d*L*_SEI_/d*t*,

(22) $$\frac{dL_{\mathrm{SEI}}}{dt}=-\frac{V_{\mathrm{SEI}}}{F}j_{\mathrm{SEI}}$$

with the mean molar volume of SEI components *V*_SEI_. Based on Equation (22), we proceed analyzing the growth behavior of the SEI with respect to the elapsed time *t*. To this aim, we insert *j*_SEI_ [see Eq. (19)], into the growth rate d*L*_SEI_/d*t* [see Eq. (22)]. In the following, we derive analytic solutions of the resulting differential equation for three different limiting cases. We compare them with the full numeric solution in the results and discussion sections.

First, if the SEI is thin, that is, *L*_app_≪*L*_diff_, we can simplify the SEI current to Equation (23),

(23) $$j_{\mathrm{SEI}}=-j_{\mathrm{SEI},0,0}e^{-\alpha_{\mathrm{SEI}}{\tilde{\eta }}_{\mathrm{SEI}}}.$$

Thus, in this regime, SEI growth is limited by the formation reaction of neutral lithium atoms. Inserting Equation (23) into the SEI‐growth Equation (22) yields a linear SEI growth with time,

(24) $$L_{\mathrm{SEI}}=\frac{V_{\mathrm{SEI}}}{F}j_{\mathrm{SEI},0,0}e^{-\alpha_{\mathrm{SEI}}{\tilde{\eta }}_{\mathrm{SEI}}}\cdot t.$$

Second, if $L_{\mathrm{diff}}\ll L_{\mathrm{app}}\ll L_{\mathrm{mig}}$ , we get

(25) $$j_{\mathrm{SEI}}=-\frac{c_{\mathrm{Li},0}DF}{L_{\mathrm{SEI}}}e^{-{\tilde{\eta }}_{\mathrm{SEI}}}.$$

Here, diffusion of neutral lithium atoms limits SEI growth, which results in a SEI growth proportional to √*t*,

(26) $$L_{\mathrm{SEI}}=L_{\mathrm{tun}}+\sqrt{2V_{\mathrm{SEI}}c_{\mathrm{Li},0}De^{-{\tilde{\eta }}_{\mathrm{SEI}}}\cdot t+{\left(L_{\mathrm{SEI},0}-L_{\mathrm{tun}}\right)}^{2}}$$

This form of SEI current and growth coincides with the form derived by Single et al.^31^ in the case of battery storage, that is, *η*_int_=0. For battery operation, *η*_int_ affects $\tilde{\eta }$_SEI_ according to Equation (18), which accelerates SEI growth during charging and decelerates SEI growth during discharging.

Third, if *L*_mig_≪*L*_app_, the SEI current has the form shown in Equations (27a)a) and (27b)b),

(27a) $$j_{\mathrm{SEI}}=\frac{c_{\mathrm{Li},0}DF^{2}j_{\mathrm{int}}}{2RT\kappa_{Li^{+},\mathrm{SEI}}}e^{-{\tilde{\eta }}_{\mathrm{SEI}}}\;\;\;\;\;\mathrm{charging},$$

(27b) $$j_{\mathrm{SEI}}=0\;\;\;\;\;dis\mathrm{charging}.$$

In this regime, migration of electrons through the SEI becomes dominant. SEI formation is irreversible, so that the SEI current must be negative. Thus, we have to distinguish between charging and discharging in this case. While SEI growth is fully suppressed during discharging, Equation (28) describes growth during charging.

(28) $$L_{\mathrm{SEI}}=\frac{V_{\mathrm{SEI}}c_{\mathrm{Li},0}DFj_{\mathrm{int}}}{2RT\kappa_{Li^{+},\mathrm{SEI}}}e^{-{\tilde{\eta }}_{\mathrm{SEI}}}\cdot t.$$

## Numerical Methods

We briefly summarize the implementation of our model developed in the previous section before we simulate SEI growth during battery cycling in the following. We model galvanostatic battery operation and thus apply a constant current density *j*, which leads to the intercalation current density *j*_int_=*j*−*j*_SEI_ according to Equation (3), with *j*_int_ affecting the lithium concentration inside the anode *c*_s_ according to the differential Equation (12). Thereby, also *U*_0_ changes according to the *U*_0_(*c*_s_)‐curve measured by Attia et al.^57^ (see SI‐1 in the Supporting Information). Growth of SEI thickness is described by Equation (22) with *j*_SEI_ obtained from Equation (19). To calculate *L*_app_, we use a continuous function, which smoothes the transition between the tunneling and the diffusion–migration regime [see Eq. (SI‐5)]. Equation (12), Equation (22), and galvanostatic conditions give a differential algebraic system of equations (DAE), which simultaneously describes battery operation and SEI growth.

We iteratively solve this DAE along the elapsed time with the ordinary differential equation solver ode15s of MATLAB. The simulation stops, when it reaches the end‐of‐charge voltage *U*_1_ or the end‐of‐discharge voltage *U*_2_. We transform the current densities, given in C‐rate, to A m^−2^ using Equation (29)

(29) $$j\left[Am^{-2}\right]=\frac{Q_{s,\mathrm{nom}}}{1\;h}\cdot \frac{1}{A_{\mathrm{cb}}}\cdot j\left[C-\mathrm{rate}\right],$$

using the nominal capacity (*Q*_s,nom_). Table S1 in the Supporting Information lists the parameters of the model.

Based on the results of the DAE, we simulate the differential capacity analysis experiments of Attia et al.^57^ with Equation (30),

(30) $${\frac{dQ_{\mathrm{SEI}}}{dU_{0}}}_{\mathrm{sim}}={\frac{dQ}{dU_{0}}}_{\mathrm{sim}}-{\frac{dQ}{dU_{0}}}_{\mathrm{baseline}}$$

with the simulated differential capacity $\frac{dQ}{dU{}_{0}}$_sim_=j$\frac{dt}{dU{}_{0}}$ . We calculate the baseline differential capacity $\frac{dQ}{dU{}_{0}}$_baseline_ from *U*_0_(*c*_s_) [see Eq. (SI‐2)]. The SEI capacity per cycle *n*, *Q*_SEI_(*n*), is obtained from integration of Equation (30) over the voltage region,

(31) $$Q_{\mathrm{SEI}}\left(n\right)=\int_{U_{1}}^{U_{2}}{\frac{dQ}{dU_{0}}}_{\mathrm{sim}}\left(n\right)dU_{0}.$$

The overall charge consumption *Q*(*n*) results from Equation (31) by adding a constant intercalation capacity *Q*_s_(*j*).

## Results

In the following, we compare our derived theory with the experiments of Attia et al.^57^ on different time scales. First, we investigate the voltage and current dependence of the short‐term SEI growth. Second, we analyze the temporal evolution of SEI growth over a long period (2<*n*<1000). Third, we investigate the time dependence of SEI growth for very long times (100<*n*).

### Short‐term SEI growth

**Figure 3:**
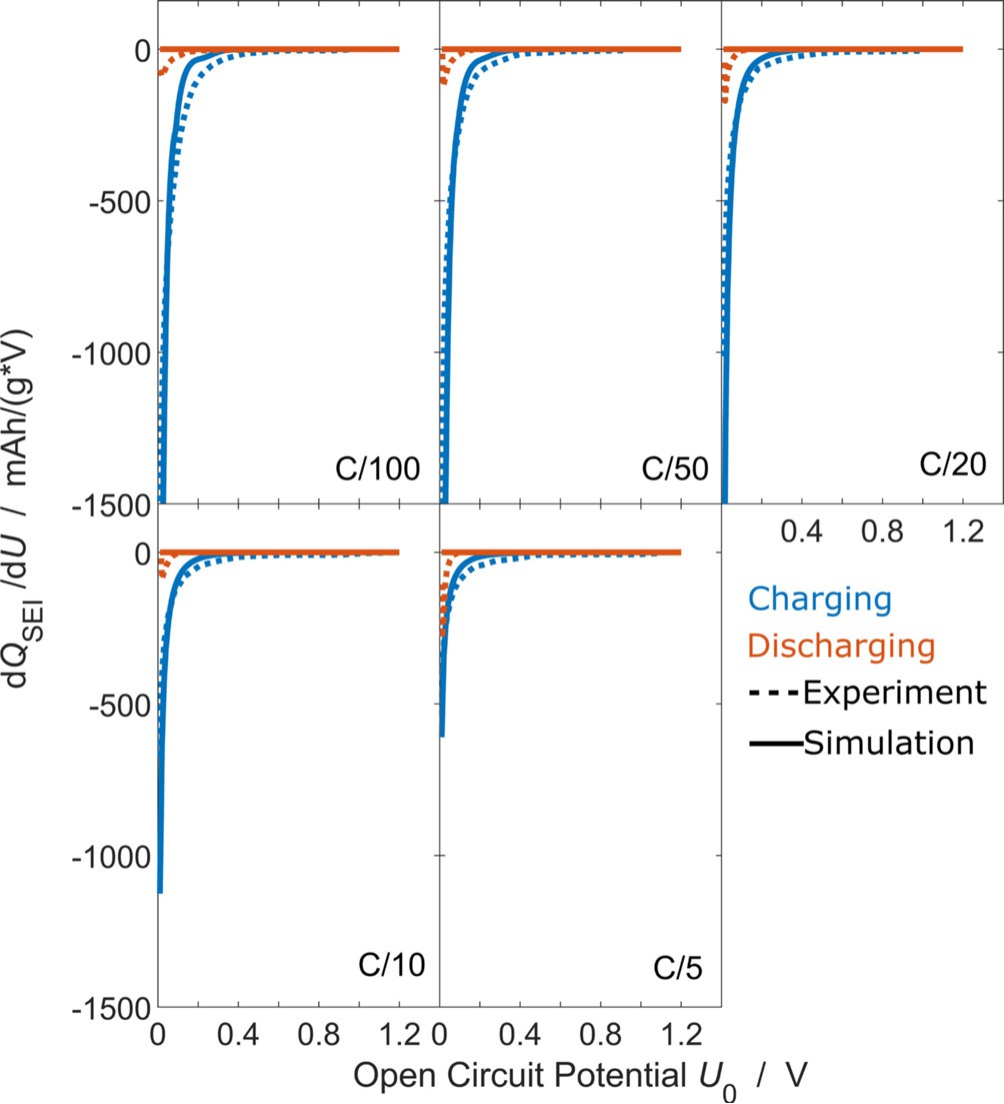
Consumed SEI capacity during the second cycle as function of OCV for different applied *j*=C/100, C/50, C/20, C/10, C/5. We compare experiments^57^ (dashed) and simulation results [solid, Eq. (30)]. Charging is depicted in blue, discharging in orange.

We compare the differential capacity analysis experiments d*Q*_SEI_/d*U*_0_ of Attia et al.^57^ with the results of our simulation in Figure 3.

The d*Q*_SEI_/d*U*_0_ curve depends exponentially on the cell voltage. Our simulations agree with this behavior for all charging currents. For discharging currents, however, we observe a deviation between experiments and simulations.

A reaction kinetic limitation causes this exponential voltage dependence. We rationalize this behavior with the approximation of the SEI current density *j*_SEI_ for thin layers in Equation (23). Inserting the definition of the Li^0^ formation overpotential $\tilde{\eta }$_SEI_ [see Eq. (18)] leads to

(32) $$j_{\mathrm{SEI}}=-j_{\mathrm{SEI},0,0}e^{-\alpha_{\mathrm{SEI}}\frac{F}{RT}\left(\eta_{\mathrm{int}}+U_{0}+\mu_{\mathrm{Li},0}/F\right)}.$$

Thus, the SEI current *j*_SEI_ depends exponentially on the OCV. The exponential factor *α*_SEI_=0.22 agrees with the experimentally determined one.

**Figure 4:**
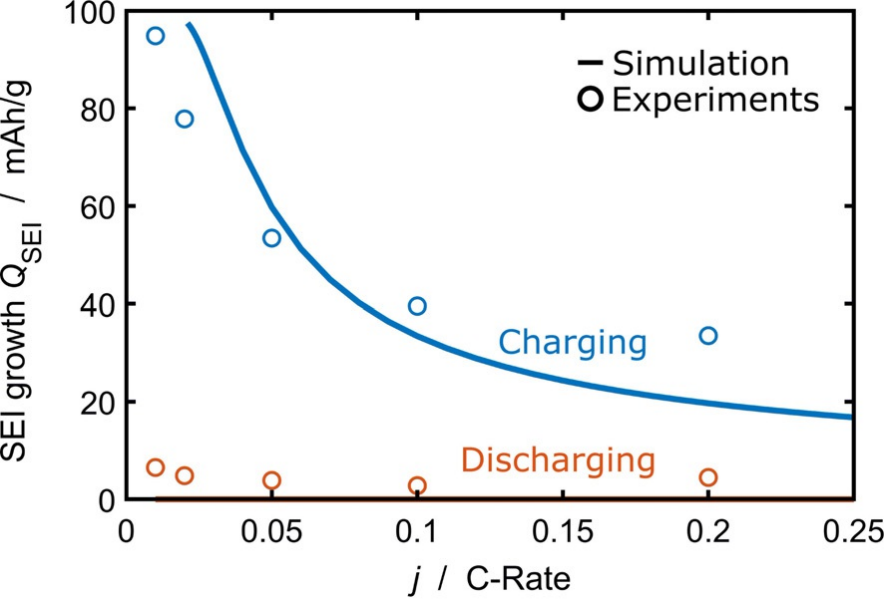
Current dependence of the overall SEI charge during the second cycle. We compare experiments^57^ (circles) and simulation results [line, Eq. (31)]. Charging is depicted in blue, discharging in orange.

The asymmetry factor *α*_SEI_ is indispensable for modeling the experimentally observed voltage dependence in Figure 4. This proofs that reaction kinetics govern the second‐cycle SEI growth. In contrast, long‐term growth models^31^ assume equilibrium at the electrode surface and are governed by the growth law in Equation (25). This growth law lacks the asymmetry factor *α*_SEI_ and thus deviates from the experiments of Attia et al.^57^ We conclude that second‐cycle SEI growth cannot be explained with equilibrium reaction conditions, but it can be explained with appropriate reaction kinetics.

The value *α*_SEI_=0.22^62^, ^63^ points to complex reaction kinetics consisting of different phenomena, which we do not resolve in our lumped Butler–Volmer kinetics in Equations (9) and (13). For example, change of electron bands at the interfaces, enhanced electron tunneling, and capacitive effects may play a role. Interestingly, in the low‐voltage regime, the OCV curve measured by Attia et al.^57^ [see Eq. (SI‐1)] shows the same exponential behavior as the SEI formation current, Equation (32). This indicates that unresolved surface processes occur.

During discharge, experiments and simulations disagree. We attribute this to a retardation effect. The experiments of Attia et al.^57^ immediately switch from charging to discharging. Thus, capacitive processes originating from the end of charging affect the discharging. Our model, however, does not resolve such capacitive processes like the lithium ion accumulation throughout the SEI. Das et al.^44^ modeled the experiments of Attia et al.^57^ Their equations described the same ideal diode effect, that is, the complete suppression of SEI current during discharge. This should suppress SEI growth during discharging, too. Furthermore, the modeling approach of Das et al.^44^ exhibited large overpotentials due to concentration polarization. In our simulations, we observe these high intercalation overpotentials, too.

Next, we analyze the influence of *j* on the total SEI growth *Q*_SEI_ in the second cycle. We determine *Q*_SEI_ by Equation (31) and compare it to the experiments of Attia et al.^57^ in Figure 4.

Our simulation results follow the experimentally measured trends. We observe a strong asymmetry between charging and discharging. During discharging, second‐cycle SEI growth is suppressed. Charging, in contrast, enhances SEI growth and *Q*_SEI_ increases with decreasing current.

Two opposing trends determine the current dependence of SEI growth per cycle during charging. On the one hand, SEI growth per cycle decreases with increasing current because the cycle time decreases according to *t*_cycle_=*c*_s,max_ *F*/*j*_int_. On the other hand, SEI growth increases with increasing current due to *η*_int_ [see Eq. (32)]. Let us calculate the dependence of *j*_SEI_ on *j*_int_. The *j*_SEI_ in Equation (32) depends on *η*_int_. We determine *η*_int_ in terms of *j*_int_ by inverting Equation (9) in the Tafel regime [see Eq. (SI‐7)]. Combining both contributions, the second‐cycle SEI capacity *Q*_SEI_ scales with *j*_int_ according to

(33) $$Q_{\mathrm{SEI}}\propto \frac{{\left(j_{\mathrm{int}}\right)}^{2\alpha_{\mathrm{SEI}}}}{j_{\mathrm{int}}}.$$

We analyze the implications of the asymmetry factor *α*_SEI_ on the observed current dependence depicted in Figure 4 based on Equation (33). For our parameter choice *α*_SEI_=0.22, we obtain a decreasing *Q*_SEI_(*j*_int_) in agreement with the experiments. The best agreement between simulation and experiment for this current dependence would be given for *α*_SEI_=0.31. This indicates that the relevant reaction mechanisms are very complex, which is not captured by our reductionist model. Bazant and co‐workers^44^, ^57^ discussed the current dependence by plotting *Q*_SEI_/*t*_cycle_ versus *j*_int_. They conclude that *Q*_SEI_/*t*_cycle_ is linear in *j*_int_, that is, *Q*_SEI_ is independent of current. This disagrees with their experimental data reprinted in Figure 4.

At small applied currents densities *j*<0.05 C, the entanglement of intercalation current and SEI current in the applied current density *j*=*j*_int_+*j*_SEI_ constitutes a fundamental challenge for modeling. Therefore, we do not plot simulation results for small current densities in Figure 4. In this case, *j*_SEI_ becomes twice as large as *j*_int_. The suppressed *j*_int_ leads to a long cycle time and a large *Q*_SEI_. Thus, at small currents, the SEI thickness crosses the critical diffusion thickness (*L*_app_>*L*_diff_) during the second cycle and diffusion dominates SEI growth [see Eq. (25)]. This leads to an increasing course of *Q*_SEI_∝(*j*_int_)^2^/*j*_int_. To sum up, for *j*≲0.05 C, our simulation results deviate from the scaling law in Equation (33).

This deviation results from our method of electron counting. Our model relies on the idea that electron consumption for SEI growth and intercalation occur simultaneously. This assumption leads to Equations (1) and (2) as well as the relationship *j*=*j*_int_+*j*_SEI_. In reality, however, Li^0^ can also be created indirectly from intercalated lithium Li_*x*_C_6_ according to Equation (34),

(34) $$\mathrm{Li}{}_{x}C{}_{6}\vec{\leftarrow }x\;\mathrm{Li}{}^{0}+C{}_{6}.$$

In this approach, the intercalation current would equal the applied current *j*=*j*_int_, so that intercalation would not be suppressed even for low *j*. However, to keep our model as simple as possible, we neglect this option for Li^0^ formation.

### Long‐term SEI growth

**Figure 5:**
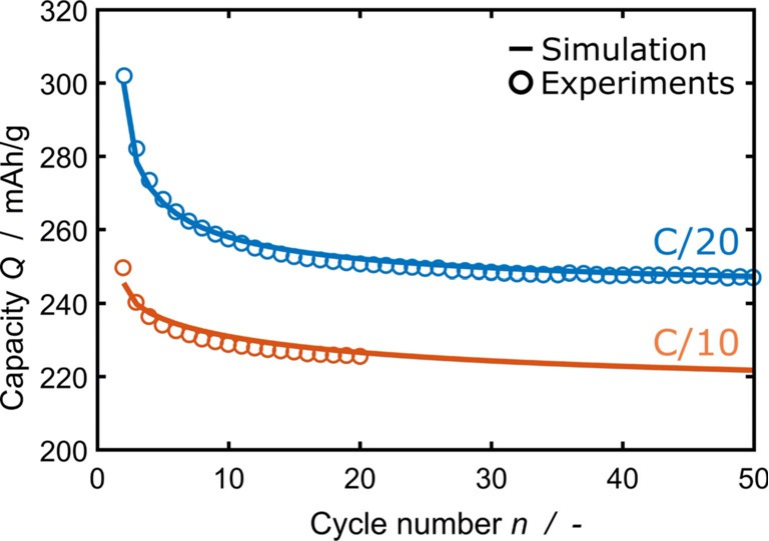
Development of the overall charge consumed for SEI formation over several cycles. We compare experiments^57^ (circles) and simulation results [solid lines, Eq. (31)]. C/20 in blue, C/10 in orange.

We continue to analyze *Q*_SEI_ and how it evolves with increasing cycle number. Figure 5 compares the simulation results for the overall capacity *Q*(*n*) determined by Equation (31) with the experiments of Attia et al.^57^ We observe that the consumed capacity decreases with each cycle and that the simulation nicely fits the experiment. Comparing the different applied current densities, we notice that *Q*_SEI_ decreases faster for C/20 compared to C/10.

The observed decrease in *Q*_SEI_ per cycle stems from transport‐limited SEI growth. In this regime, our model agrees with the model for neutral lithium diffusion of Single et al.^31^ Thus, our model predicts the well‐known square‐root‐of‐time dependence of the overall SEI growth *L*_SEI_ [see Eq. (26)], for long times.

Based on the growth law in this limit [see Eq. (26)], we derive the dependence of SEI growth *Q*_SEI_(*n*) on *n*. To this aim, we link *n* to the elapsed time *t*=*Q*_s,max_/*j*_int_⋅*n* and the overall SEI charge consumption *Q*_SEI,tot_ to the SEI thickness *L*_SEI_=*V*_SEI_/*F*⋅*Q*_SEI,tot_. Taking the derivative of *L*_SEI_ with respect to *n* [see Eq. (26)] yields the capacity fade per cycle,

(35) $$Q_{\mathrm{SEI}}\left(n\right)\;=\frac{dQ_{\mathrm{SEI},\mathrm{tot}}}{dn}=\left[\frac{V_{\mathrm{SEI}}}{2c_{\mathrm{Li},0}DF^{2}}\cdot \frac{j_{\mathrm{int}}}{e^{-{\tilde{\eta }}_{\mathrm{SEI}}}}\cdot n\right.{\left.+{\left(\frac{F(L_{\mathrm{SEI},0}-L_{\mathrm{tun}})}{V_{\mathrm{SEI}}}\right)}^{2}\right]}^{-1/2}.$$

Thus, *Q*_SEI_(*n*) decays monotonously with the inverse of the cycle number as 1/√*n*. The slope depends on the current density in the form $j_{\mathrm{int}}/e^{-F\eta_{\mathrm{int}}/RT}\approx 1/j_{\mathrm{int}}$ [see Eqs. (18) and (SI‐7)] and is thus larger for C/20 than for C/10.

### Ultra long‐term SEI growth

**Figure 6:**
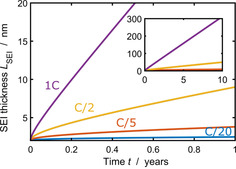
*L*_SEI_ with respect to *t* for continuous cycling of graphite in a state‐of‐charge range of 0.2≤*c*_s_/*c*_s,max_≤0.8 for different applied *j*=C/20, C/5, C/2,1 C.

We proceed by analyzing the SEI growth for very long times (100<*n*). In Figure 6, we show the growth of SEI thickness *L*_SEI_ over time for continuous cycling of a graphite anode at various *j* (see Table SI‐2).^64^, ^65^, ^66^ We observe that the SEI thickness grows faster for higher charging currents. Additionally, the slope of the curves changes with time, starting from a square‐root‐of‐time dependence and shifting towards a linear time dependence.

SEI growth is faster for higher currents because the SEI current increases with *j*_int_ according to Equations (25) and (27). The cause for the transition in time dependence is a shift from diffusion‐limited to migration‐limited growth. Over time, *L*_SEI_ grows and approaches *L*_mig_. Below the transition thickness, diffusion limits SEI growth according to Equation (26) leading to a square‐root‐of‐time dependence. Above the transition thickness, electromigration is the growth‐limiting process, which results in a linear time dependence of the curve, according to Equation (28).

A shift to linear SEI growth was so far observed experimentally by different groups.^45^, ^51^, ^56^, ^67^, ^68^ This transition is typically attributed to mechanical effects, for example, repeated SEI fracture and regrowth.^32^, ^45^, ^69^ Our approach shows a complementary explanation of linear SEI growth within electrochemistry.

## Discussion

In the previous section, we revealed that different growth mechanisms dominate at different time scales. We follow this line of thought in this section and systematically analyze the transition between the growth regimes. We analyze the transition between short‐term, long‐term, and ultra long‐term SEI growth and describe the corresponding correlation between current, voltage, and time dependence. We first calculate the SEI current magnitude depending on the operating conditions and study the asymmetry between charging and discharging. Subsequently, we analyze the influence of operating conditions on the transition between the regimes. First, we investigate the transition from reaction to diffusion limitation. Second, we look at the transition between diffusion and electromigration limitation.

### Asymmetry between charging and discharging

We analyze how the operating conditions influence the SEI growth rate d*L*_SEI_/d*t*. To this aim, we take a look at the growth rate for various *j* and OCVs with an SEI thickness of *L*_SEI_=3 nm.

**Figure 7:**
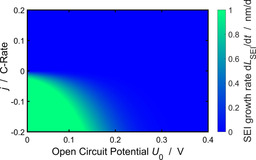
SEI growth rate with respect to applied current and OCV for an SEI tickness of *L*_SEI_=3 nm [see Eq. (22)].

Figure 7 clearly shows the asymmetry between charging and discharging: SEI grows fast during charging and slow during discharging. Furthermore, low electrode voltages accelerate SEI growth. Both trends result from the SEI overpotential $\tilde{\eta }$_SEI_ [Eq. (18)], which exponentially increases the SEI current for low voltages and high intercalation currents. These results show that the capacity of lithium‐ion batteries fades fastest for high state‐of‐charge and high charging rate.

### Transition between regimes

We proceed by identifying the different dominant growth mechanisms based on the respective time dependence of SEI growth, *L*_SEI_(*t*). To this aim, we express the scaling of SEI thickness with time in the general form shown in Equation (36),

(36) $$L_{\mathrm{SEI}}\propto t^{\beta }\Leftrightarrow \beta =\frac{d\log(L_{\mathrm{SEI}})}{d\log(t)}.$$

The parameter *β* indicates the dominant growth mechanism according to

‐: *β*=1: reaction limitation or migration limitation during charging,
‐: *β*=0.5: diffusion limitation,
‐: *β*=0: migration limitation during discharging.

**Figure 8:**
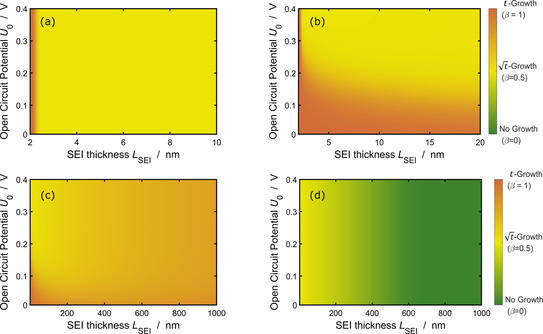
Scaling factor *β* [see Eq. (36)] of time dependence of SEI growth as a function of OCV and SEI thickness according to Equation (22). Red indicates reaction limitation [Eq. (23), *β*=1] or migration limitation during charging [Eq. (27a), *β*=1]. Yellow indicates diffusion limitation [Eq. (25), *β*=0.5] and green migration limitation [Eq. (27b), *β*=0). (a) Battery storage. (b) Battery charging with *j*=−0.2 C in the short‐term evaluation. (c) Battery charging and (d) battery discharging with *j*=−0.2 C in the long‐term evaluation.

*β* depends on the applied *j*, the OCV, and *L*_SEI_. In a recent study, Attia et al.^57^ discussed the deviation of the scaling coefficient from the literature standard *β*=0.5 in cell‐aging experiments. First, we look at the growth behavior during storage in Figure 8 a. We observe a sharp transition between reaction and diffusion limitation for *L*_SEI_≈2.4 nm, which is independent of the OCV. *L*_tun_ is the reason for this transition. Below this thickness, electrons easily tunnel through the SEI, so that the SEI formation is limited by the Li^0^ reaction kinetics. Above this thickness, diffusion through the SEI becomes dominant, leading to a transport limitation in agreement with the measurements of Keil et al.^67^ and the model of Single et al.^31^ During battery charging (see Figure 8 b), the transition between reaction and diffusion limitation is smeared out. We observe in Figure 8 b that SEI growth is reaction limited for a thin SEI and a low OCV. Diffusion limits growth for a high OCV and a thick SEI.

To understand this behavior, we recall the premise for reaction limitation derived in the Theoretial Background section, *L*_app_≪*L*_diff_. This is fulfilled for low SEI thicknesses or large critical diffusion thicknesses. According to Equation (20), *L*_diff_ grows exponentially with decreasing Li^0^ formation overpotential $\tilde{\eta }$_SEI_ and thereby with decreasing OCV [see Eq. (18)]. We thus observe reaction limitation for low OCVs and low *L*_SEI_.

The transition from reaction to diffusion limitation has important implications for the current, OCV, and time dependence of SEI growth [see Eqs. (23) and (25)]. For reaction‐limited SEI growth, the SEI thickness scales with *t*; for diffusion‐limited SEI growth, it scales with √*t*. OCV and current dependence are weaker for reaction limitation due to the exponential factor *α*_SEI_. Reaction limitation exhibits an exponential dependence on OCV, weakened by *α*_SEI_, and a sublinear dependence on *j*. In contrast, transport limitation shows an exponential dependence on OCV and a quadratic current dependence.

Next, we analyze the growth behavior of the SEI for longer times in Figures 5 c and d. We observe a continuous transition from diffusion (yellow) to migration (red in panel (c) and green in panel (d)) limitation for all voltages.

This transition arises as *L*_app_ approaches *L*_mig_, defined by Equation (21). This shift in limiting mechanism leads to a shift in the time dependence of SEI thickness from √*t* to *t* (during charging) respective no growth (during discharging) according to Equations (25) and (27). We note that the current dependence is stronger for migration limitation.

Summarizing Figure 8 b, c and d, we observe a transition in the time dependence of SEI growth from *t*→√*t*→*t*/(const.) due to a shift in the dominant formation mechanism from reaction to diffusion to migration limited. This finding explains phenomenologically derived models for the capacity fade Δ*Q* of the form

(37) $$\Delta Q\propto t^{\beta }\;\;\;\;0\leq \beta \leq 1,$$

as transition between either diffusion and reaction or diffusion and migration limitation.^49^, ^50^, ^55^ Moreover, our findings show that linear capacity fade is inherent to the electrochemistry of the system and not necessarily caused by SEI fracture and reformation.^32^, ^45^, ^68^, ^69^

## Conclusions

We have extended an existing model for solid–electrolyte interphase (SEI) growth during battery storage^31^ to incorporate the effects of battery operation. A comparison of the model predictions with the experiments of Attia et al.^57^ showed very good agreement. Based on the thus validated model we proceed analyzing the SEI growth behavior in detail. We find that the formation reaction of neutral lithium atoms initially limits SEI growth. With increasing SEI thickness, first diffusion and then electromigration of the electrons coordinated to lithium ions limits further SEI growth. The resulting model for diffusion limitation agrees with the model of Single et al.^31^ in the case of battery storage.

Our novel modeling approach predicts a shift in time (*t*) dependence of capacity fade from *t*→√*t*→*t*/const. over time. For the first time, this time dependence explains the so far empirically motivated capacity fade Δ*Q* equations of the form Δ*Q*∝*t* ^*β*^ with the scaling coefficent *β*, 0≤*β*≤1, as transitions between transport‐ and reaction‐limited growth.^41^, ^49^, ^50^, ^55^ Moreover, these new insights show that besides SEI fracture and reformation the inherent electrochemistry of SEI growth leads to a linear SEI growth during long‐term battery cycling, too.

Our theory can be extended to account for lithium plating, that is, the precipitation of neutral lithium atoms Li^0^ at the anode, as we model Li^0^ as mediator for SEI growth. The amount of Li^0^ in the SEI exponentially increases at low potentials when lithium plating occurs. We hypothesize that this increase in lithium amount could act as seed for lithium plating. In turn, inhomogeneously plated lithium would affect charge transfer through the SEI. To resolve inhomogeneous SEI growth and lithium plating caused by locally varying operating conditions, the theory developed in this work can be implemented into a three‐dimensional battery simulation.

## Conflict of interest

*The authors declare no conflict of interest*.

## Supporting information

As a service to our authors and readers, this journal provides supporting information supplied by the authors. Such materials are peer reviewed and may be re‐organized for online delivery, but are not copy‐edited or typeset. Technical support issues arising from supporting information (other than missing files) should be addressed to the authors.

Supplementary
