# Supplementary material for: Solid–Electrolyte Interphase During Battery Cycling: Theory of Growth Regimes
Source: ChemSusChem. 2020 Jun 29;13(15):3901–10. doi: 10.1002/cssc.202000867 (PMC7496968; doi:10.1002/cssc.202000867)
Supplement: Supplementary file 1 — Supplementary [file CSSC-13-3901-s001.pdf]

ChemSusChem

Supporting Information

Solid–Electrolyte Interphase During Battery Cycling: Theory of Growth Regimes

Lars von Kolzenberg,[a, b] Arnulf Latz,[a, b, c] and Birger Horstmann*[a, b, c]

I. PARAMETRIZATION

State of charge c_s and open circuit voltage U_0 are related by the OCV-curve measured by Attia et al. [1] (see figure 3c in their work). We fit the curve with expression SI-1,

$$U_0 = \left(\frac{1.24 - c_\mathrm{s}/c_\mathrm{s,max}}{1.16}\right)^{2.92} \mathrm{V} \tag{SI-1}$$

with the maximum concentration $c_\mathrm{s,max} = Q_\mathrm{s,max}\rho_\mathrm{cb}/F$. Based on this relationship between charge and voltage, we calculate the baseline cycle for differential capacity analysis, $\frac{\mathrm{d}Q}{\mathrm{d}U_0}_\mathrm{baseline}$. For this, we rearrange equation SI-1 as $c_\mathrm{s}(U_0)$ and then take the derivative with respect to voltage. Additionally, we replace c_s with $Q_\mathrm{s}\rho_\mathrm{cb}/F$ and $c_\mathrm{s,max}$ with $Q_\mathrm{s,max}\rho_\mathrm{cb}/F$.

$$\frac{\mathrm{d}Q}{\mathrm{d}U_0}_\mathrm{baseline} = -313.5\,\mathrm{C/g/V}^{0.66} U_0^{-0.66} \tag{SI-2}$$

The OCV-curve can furthermore be subdivided into a linear part at high potentials (equation SI-3) and an exponential part at low potentials (equation SI-4).

$$Q_\mathrm{lin} = 129.62\mathrm{mA\,h\,g^{-1}} - 125.17\mathrm{mA\,h\,g^{-1}\,V^{-1}} \cdot V \tag{SI-3}$$

$$Q_\mathrm{exp} = 93.27\mathrm{mA\,h\,g^{-1}} \cdot \exp\left(-0.21\frac{FU_0}{RT}\right) \tag{SI-4}$$

We account for tunneling of electrons inside the SEI by introducing an apparent thickness $L'_\mathrm{app} = L_\mathrm{SEI} - L_\mathrm{tun}$. However, if the SEI is thinner than the typical tunneling range, $L_\mathrm{SEI} < L_\mathrm{tun}$, the apparent thickness becomes negative. We avoid this nonphysical behavior by introducing the continuous function SI-5,

$$L_\mathrm{app} = \frac{L'_\mathrm{app}}{2} + \sqrt[10]{\left(\frac{L'_\mathrm{app}}{2}\right)^{10} + L^2_\mathrm{app,0}}. \tag{SI-5}$$

Thereby, we employ a smooth transition between tunneling and diffusion limited growth with the limiting cases $L_\mathrm{app} \to 0$ for $L_\mathrm{SEI} < L_\mathrm{tun}$ and $L_\mathrm{app} = L_\mathrm{SEI} - L_\mathrm{tun}$ for $L_\mathrm{SEI} > L_\mathrm{tun}$.

We list the parameter of the model in table SI-1.

Variable	Description	Value	Unit	Source
U_1	Maximum Voltage during cycling	1.2	V	[1]
U_2	Minimum Voltage during cycling	0.01	V	[1]
$Q_{\mathrm{s,nom}}$	Nominal maximum capacity of carbon black	200	$\mathrm{mA\,h\,g^{-1}}$	[1]
$Q_{\mathrm{SEI,0}}$	Irreversible capacity loss of first cycle	310	$\mathrm{mA\,h\,g^{-1}}$	[1]
$L_{\mathrm{SEI,0}}$	SEI growth of first cycle	2	nm	[2]
j	Intercalation current	2-40	$\mathrm{mA\,g^{-1}}$	[1]
$A_{\mathrm{cb,m}}$	Mass specific area of carbon black	62	$\mathrm{m^2\,g^{-1}}$	[3]
ρ_{cb}	Density of carbon black	1.4	$\mathrm{g\,cm^{-3}}$	[4]
A_{cb}	Volume specific area of carbon black	86.8×10^6	$\mathrm{m^2\,m^{-3}}$	$A_{\mathrm{cb,m}} \cdot \rho_{\mathrm{cb}}$
$Q_{\mathrm{s,max}}$	Maximum capacity of carbon black	218	$\mathrm{mA\,h\,g^{-1}}$	[1]
$c_{\mathrm{s,max}}$	Maximum lithium concentration of carbon black	3.16	$\mathrm{mol\,L^{-1}}$	$Q_{\mathrm{s,max}} \cdot \rho_{\mathrm{g}}/F$
V_{SEI}	Mean molar volume of SEI species	1.078×10^{-5}	$\mathrm{m^3\,mol^{-1}}$	$L_{\mathrm{SEI,0}}A_{\mathrm{cb,m}}F/Q_{\mathrm{SEI,0}}$
D_{Li}	Diffusion coefficient of Li atoms inside the SEI	1×10^{-15}	$\mathrm{m^2\,s^{-1}}$	Assumed
$c_{\mathrm{Li,0}}$	Reference concentration of Li atoms at the anode-SEI interface	1	$\mathrm{mol\,L^{-1}}$	Assumed
$\kappa_{\mathrm{Li^+,SEI}}$	Lithium ion conductivity of the SEI	1×10^{-8}	$\mathrm{S\,m^{-1}}$	Assumed
$j_{0,0}$	Butler-Volmer rate constant for intercalation	6.4×10^{-7}	$\mathrm{A\,m^{-2}}$	Fitted
$j_{\mathrm{SEI,0,0}}$	Butler-Volmer rate constant for Li atom formation	7.04×10^{-5}	$\mathrm{A\,m^{-2}}$	Fitted
F	Faraday's constant	96485	$\mathrm{C\,mol^{-1}}$	
R	Universal gas constant	8.314	$\mathrm{J\,mol^{-1}\,K^{-1}}$	
T	Temperature	303	K	[1]
α_{SEI}	Symmetry factor for Butler-Volmer	0.22	-	[5]
$\mu_{\mathrm{Li,0}}$	Chemical reference potential of Lithium interstitial atoms inside the SEI	17.4	$\mathrm{kJ\,mol^{-1}}$	Fitted
$L_{\mathrm{app,0}}$	Reference apparent thickness for equation SI-5	0.05	nm	Fitted
L_{tunnel}	Typical tunneling distance for electrons	2.05	nm	Fitted

Table SI-1: List of model parameters for carbon black.

Variable	Description	Value	Unit	Source
$A_{\mathrm{g,m}}$	Mass specific area of graphite	0.6	$\mathrm{m^2\,g^{-1}}$	[6]
ρ_{g}	Density of graphite	2	$\mathrm{g\,cm^{-3}}$	[4]
A_{g}	Volume specific area of graphite	1.2×10^6	$\mathrm{m^2\,m^{-3}}$	$A_{\mathrm{g,v}} \cdot \rho_{\mathrm{g}}$
$Q_{\mathrm{s,max,g}}$	Maximum capacity of graphite	372	$\mathrm{mA\,h\,g^{-1}}$	[7]
$c_{\mathrm{s,max,g}}$	Maximum lithium concentration of graphite	7.71	$\mathrm{mol\,l^{-1}}$	$Q_{\mathrm{s,max,g}} \cdot \rho_{\mathrm{g}}/F$

Table SI-2: List of model parameters for graphite. Parameters not listed are equal to the carbon black parametrization shown in table SI-1

II. TAFEL APPROXIMATION

For large overpotentials η_{int}, we can approximate the Butler-Volmer equation 9 with the Tafel equation SI-6 ("+" for deintercalation, "-" for intercalation)

$$j_{\mathrm{int}} = \pm 2 j_0 e^{\pm \frac{F}{2RT}\eta_{\mathrm{int}}}. \tag{SI-6}$$

Using expression SI-6, we reformulate the exponential dependence of equation 23 on η_{int} as shown in equation SI-7,

$$e^{-\alpha_{\mathrm{SEI}}\frac{F}{RT}\eta_{\mathrm{int}}} = \left(\frac{j_{\mathrm{int}}}{2j_0}\right)^{2\alpha_{\mathrm{SEI}}} \propto j_{\mathrm{int}}^{2\alpha_{\mathrm{SEI}}}. \tag{SI-7}$$

[1] P. M. Attia, S. Das, S. J. Harris, M. Z. Bazant, W. C. Chueh, *J. Electrochem. Soc.* **2019**, *166*, E97–E106, doi:10.1149/2.0231904jes.

[2] W. Huang, P. M. Attia, H. Wang, S. E. Renfrew, N. Jin, S. Das, Z. Zhang, D. T. Boyle, Y. Li, M. Z. Bazant, B. D. McCloskey, W. C. Chueh, Y. Cui, *Nano Lett.* **2019**, *19*, 5140–5148, doi:10.1021/acs.nanolett.9b01515.

[3] S. Das, P. M. Attia, W. C. Chueh, M. Z. Bazant, *J. Electrochem. Soc.* **2019**, *166*, E107–E118, doi:10.1149/2.0241904jes.

[4] J. J. Kipling, J. N. Sherwood, P. V. Shooter, N. R. Thompson, *Carbon* **1964**, *1*, 321–328, doi:10.1016/0008-6223(64)90286-6.

[5] Y. Li, Y. Qi, *Energy Environ. Sci.* **2019**, 1286–1295, doi:10.1039/c8ee03586e.

[6] O. N. Shornikova, E. V. Kogan, N. E. Sorokina, V. V. Avdeev, *Russ. J. Phys. Chem. A* **2009**, *83*, 1022–1025, doi:10.1134/S0036024409060260.

[7] J. M. Tarascon, M. Armand, *Mater. Sustainable Energy Appl.* **2010**, *414*, 171–179, doi: 10.1142/9789814317665_0024.
